# Developing new ways to listen: the value of narrative approaches in empirical (bio)ethics

**DOI:** 10.1186/s12910-021-00691-7

**Published:** 2021-09-16

**Authors:** Bernadette Roest, Megan Milota, Carlo Leget

**Affiliations:** 1 University of Humanistic Studies, Kromme Nieuwegracht 29, 3512 HD Utrecht, The Netherlands; 2 Julius Center for Health Sciences and Primary Care, University Medical Center Utrecht, P.O. Box 85500, 3508 GA Utrecht, The Netherlands

**Keywords:** Empirical bioethics, Methodology, Narrative approaches

## Abstract

The use of qualitative research in empirical bioethics is becoming increasingly popular, but its implementation comes with several challenges, such as difficulties in aligning moral epistemology and methods. In this paper, we describe some problems that empirical bioethics researchers may face; these problems are related to a tension between the different poles on the spectrum of scientific paradigms, namely a positivist and interpretive stance. We explore the ideas of narrative construction, ‘genres’ in medicine and dominant discourses in relation to empirical research. We also reflect on the loss of depth and context that may occur with thematic or content analyses of interviews, and discuss the need for transparency about methodologies in empirical bioethics. Drawing on insights from narrative approaches in the social sciences and the clinical-educational discipline of Narrative Medicine, we further clarify these problems and suggest a narrative approach to qualitative interviewing in empirical bioethics that enables researchers to ‘listen (and read) in new ways’. We then show how this approach was applied in the first author’s research project about euthanasia decision-making. In addition, we stress the important *ethical* task of scrutinizing methodologies and meta-ethical standpoints, as they inevitably impact empirical outcomes and corresponding ethical judgments. Finally, we raise the question whether a ‘diagnostic’, rather than a ‘problem-solving’, mindset could and should be foregrounded in empirical ethics, albeit without losing a commitment to ethics’ normative task, and suggest further avenues for theorizing about listening and epistemic (in)justice in relation to empirical (bio)ethics.

## Background

How are qualitative interviews typically conducted in bioethics research? And how might narrative approaches^1^ in the social sciences, humanities and clinical education enrich this research practice? These questions constitute the foundation and impetus of this article. We pose these questions because qualitative methods—in particular, face-to-face interviews and focus-groups—have become increasingly popular amongst bioethics-scholars in the last decades [5, 6]. That being said, the implementation of these methods in empirical bioethics^2^ can be challenging. This is due, in part, to difficulties aligning (moral) epistemologies and methods. Problems can also arise when determining if and how to distinguish facts and values. In addition, researchers must choose from a concordant multitude of methodologies and may be insufficiently trained in empirical methods traditionally used in the social sciences [5–10].

As academics working at the intersection of clinical practice, education and research in bioethics, we have personal experience with many of these challenges. We have found that qualitative methods for gathering and analyzing data in one field aren’t always accepted in another. Standards for assessing quality, evaluating research ethical-issues and reporting on findings can also be vastly disparate [11, 12]. A qualitative study of focus groups published in a linguistically-oriented journal looks very different from a focus group study in a biomedical journal, for instance. Even the ways in which qualitative methods are taught to students and researchers varies between faculties and fields. Meanwhile, we also observed a need to broaden our toolkit of qualitative methods in empirical bioethics, to obtain more detailed and contextualized accounts of illness and caring experiences that seem vital to understand and ethically assess complex healthcare practices.

While searching for robust and transparent methodologies that could address the above mentioned challenges, we actively deepened our affiliations with narrative approaches and Narrative Medicine.^3^ These engagements gradually taught us to ‘listen (and read) in new ways’. They enriched our understanding of qualitative research practices and simultaneously changed our teaching and clinical practice. Meeting regularly from 2018 till date to discuss theoretical backgrounds and possible applications, we attempted to ‘translate’ valuable skills and insights from these disciplines to empirical bioethics.

In this article, we will share some of our insights into our version of ‘narrative thinking’. In particular, we describe a narrative approach to qualitative interviewing with illustrative examples from the first author’s research project on euthanasia decision-making in Dutch general practice. We hope this detailed exposition will prove helpful for fellow-researchers in bioethics searching for methodological guidance.

Furthermore, the considerations and approaches discussed in this paper are intended to draw attention to a ‘diagnostic’ attitude in empirical bioethics, an attitude marked by a continuous re-examination of the questions we ask our research participants, the stories they tell us, and the frames we use in our inquiries. We contend that this diagnostic attitude, in contrast to a more problem-solving one, seems most appropriate should we as empirical bioethicists want to formulate ethical guidance that is more attuned to the ambiguous and nuanced lives and concrete care-practices of patients, relatives and healthcare professionals.

## A spectrum of scientific paradigms and the implications for qualitative research in bioethics

As a result of our immersion in narrative approaches and Narrative Medicine, we came to understand that conducting qualitative research in bioethics not only entails working at the intersection of different *disciplinary fields* (ethics/philosophy, social science, medicine) but also between different poles of a *spectrum of scientific paradigms,* namely positivist versus interpretivist traditions [1, 12, 17]. Contemporary medicine, the discipline most frequently collaborated with in empirical bioethics [5], mainly operates from the positivist pole of this spectrum. In contrast, most approaches in qualitative research can be found at the interpretivist pole.^4^ For empirical bioethicists conducting qualitative research, it seems necessary to recognize this spectrum of scientific paradigms in order to avoid certain pitfalls and to become aware of (and balance between) the different standards of good research that come with the different positions on the spectrum.

### Narrative construction, genres and dominant cultural narratives

When one approaches interviews from a positivist stance, the clinician or researcher may take the interview content at face value, as objective answers to objective questions posed by an interlocutor operating from a neutral position.^5^ However, scholars in narrative research operating from an interpretivist paradigm take a different perspective^6^; every qualitative interview is an encounter in which narrative construction takes place on multiple levels simultaneously. This construction is influenced by both the interviewer and participant, each with their own specific backgrounds and positions, and is always tied to a specific historical and cultural context [18–22].

Chambers points at something similar when he argues that “as narrative theorists know well (and most philosophers when pressed) there are no objective stories; all tales are told by some teller in a way that simultaneously reveals and conceals events” [23, p. 40]. He describes how medical-ethical cases—bioethicists’ objects of analysis—seem to follow a ‘genre’ with specific narrative features: action-oriented, characters lacking psychological depth and with sparse attention for historical time and life-worlds outside the clinic.

In a similar way, patients’ stories seem to take a specific shape in their encounters with clinicians; they can be disjointed, focusing on medical-technical aspects, and leaving out clues to social and cultural contexts [18, 20, 24]. Furthermore, factors such as age, gender, cultural and mental health background, expectations and power-imbalances in the doctor–patient relationship, and organizational factors have shown to profoundly influence what is being told by patients and how clinicians respond to it [25–28]. And even when a person-centered care approach is deliberately applied, some patients’ stories are taken more seriously than others [29]. On a level transcending medicine and bioethics, both social scientists and philosophers have described (and criticized) dominant cultural narratives in Western countries that focus on the autonomous self, coherence, control and continuity [30–33].

An awareness of a narrative’s form, structure and underlying ‘genre’ combined with an attention to dominant cultural discourses present in the narrative may enable an empirical bioethicist to look more critically at the process of qualitative interviewing and the results it may yield. The researcher can furthermore reflect on his or her own contribution to the construction of the interview-data and to what extent the interview questions and subsequent analysis of the data may reproduce existing ‘genres’ in medicine and bioethics. A final question worth asking is whether or not fragmented, contradictory or non-coherent stories receive sufficient attention and representation in research design, data analysis and valorization compared to more coherent stories [25].

### Breadth versus depth in the analysis of qualitative interview-data

Another point worth considering for qualitative researchers in empirical bioethics, is how a positivist or interpretive outlook influences the analysis of interview data. Content analysis and thematic analysis—admittedly valuable and widely applicable approaches—are the most popular techniques for analyzing qualitative interviews among empirical bioethicists [5]. However, these approaches aim at generating overarching themes across multiple interviews and therefore run the risk of diverging into a positivist approach marked by generalization and quantification.^7^ Some scholars have criticized this approach, voicing a concern that “analyst were ignoring the meaning-making person through decomposing interview transcripts into a series of themes or discourses” [36 paraphrasing 19].

In contrast, narrative researchers often focus in their analysis on “*how* and *why* a particular event is storied” [21, p. 12], emphasis added. In other words, they explore who is able to speak about certain themes and under what conditions; they also focus on the particularities and context of the stories told. If thematic analysis is subsequently applied in narrative research, it focuses on the emergence and sequencing of themes *inside* a specific interview instead of *across* interviews [21]. This “case-centered commitment” constitutes the most significant difference between narrative methods and other “category-centered models” of qualitative research [21, p. 12, 74] .

The analytical arsenal for narrative researchers extends far beyond the aforementioned ‘thematic narrative analysis’. In health psychology, medicine and ethics, scholars have studied ‘storyline elements’ or ‘narrative features’. Examples include voice, teller, characters, place, time, acts or events, breaches, ‘peripeteia’ (i.e. sudden changes), mood, metaphor, purpose and plot [14, 21, 23, 36–38]. Another point of focus for narrative researchers are the different levels of narrative construction, namely the personal-autobiographical, the dialogical-interpersonal and societal-cultural level [20, 39]. Other scholars have also applied structural analysis [21, 40] or have drawn inspiration from socio-linguistics and ethnomethodological approaches like conversation analysis and discourse analysis in their analyses of narrative interviews [40, 41].^8^

The choice in analytic approach always depends on the research aim and question. However, a specific challenge seems to be finding analytical approaches that foster an in-depth inquiry of interview data while simultaneously yielding results that are deemed relevant to the ethical issues and clinical practices being scrutinized. An analysis of interview data focusing on the particularities of language and organization of stories may at first cause medical practitioners and clinical bioethicist to exclaim ‘So what?’. However, we contend that certain research aims and questions require a more thorough and in-depth analysis and that approaches from the field of narrative research provide the much needed additions to the current toolbox of analytical approaches that bioethicists commonly employ.

### Stringent methodological guidelines and ‘interpretive magic’

The tension between the positivist and interpretivist traditions can be found in discussions about the need for stringent methodological frameworks in qualitative research in empirical bioethics. Some scholars have warned against too much standardization of qualitative research as it could undermine approaches that rely heavily on researchers’ involvement in the data collection process. Standardization could also diminish or prohibit one of the fundamental aspects of qualitative research: the variation in interpretations [42, 43].

Researchers working in an environment dominated by positivist traditions or world views run the risk of having their findings dismissed as subjective and non-generalizable. In the worst case, results may be derided as a form of ‘interpretive magic’, even when the research project in question has followed the quality-criteria connected to that specific research practice and the related scientific paradigm. As scholars who have studied and worked in both medical and humanities faculties, we understand both sides of this debate. A strategy that has worked for us is to become acquitted with and refer to existing guidelines for conducting and reporting qualitative research in order to facilitate transparency and study replicability [7, 10, 44, 45].

## A narrative approach to qualitative interviewing and analysis in empirical bioethics: fostering close-listening and close-reading

As a result of our conversations with each other, our own experiences as researchers and educators, and our Narrative Medicine training, we have attempted to develop an approach for conducting qualitative interview studies in empirical bioethics that is informed by the insights of narrative research and narrative medicine. The approach is aimed at 1) fostering a more in-depth understanding of the persons and practices being studied and 2) helping researchers trained in medicine and bioethics access valuable insights and techniques from interpretivist traditions. In the following section, we will first describe the approach as it was used by the first author (BR) in her research project on euthanasia decision-making in Dutch general practice. This description can serve as a guide for other researchers as well. Afterwards, we discuss some critical insights that emerged from working with this approach. These insights are meant to illustrate how ‘narrative thinking’ can further enrich our understanding of qualitative research projects in bioethics.

### A narrative approach to qualitative interviewing and analysis

BR’s research project on euthanasia decision-making in Dutch general practice was inspired by a discrepancy she observed as a GP between the close involvement of family members in euthanasia decision-making in practice, versus the silence about their position in legislation and guidelines [46]. In addition, she noticed that it seemed difficult to capture the messiness and complexity of everyday euthanasia-practices, as well as their social-political context, in empirical research and normative reflection [47, 48]. Therefore, she searched new ways to explore the Dutch practice of euthanasia and found directions in the moral epistemology of Margaret Urban Walker [33, 49]. BR has described this moral epistemological stance and the connection with narrative approaches to qualitative research in detail in an earlier publication [47].

BR formulated a twofold research-question to address both the descriptive and normative dimension of the research-project, which we consider to be deeply interrelated: (1) What can we learn from both content and form of co-constructed interview-narratives about the needs and felt responsibilities of patients suffering from cancer, their relatives and healthcare professionals during euthanasia decision-making; and (2) what impact may this knowledge have on our ethical evaluation of euthanasia decision-making in Dutch general practice?

As one can see, the research questions reflect the previously discussed ideas about narrative construction in qualitative interviews. Furthermore, in this research project a broad conception of ‘narrative’ was adopted. Following Riessman and Squire, interviews were seen as “narrative occasions” [21, p. 23]; in other words, both verbatim transcribed interviews (including non-verbal utterances)^9^ and field notes of pre- and post-interview conversations and -impressions were considered part of these narrative occasions to be analyzed [2, 21, 51]. In addition, the narratives were considered a means of representing, reconstituting and expressing experience [52], rather than unmediated, direct expressions of experience.

BR recruited patients, relatives, GPs and other healthcare professionals (minimal 10 participants per group) via purposive and snowball-sampling. For the interviews, BR employed informal, open-ended interviewing techniques rather than more rigidly organized semi-structured interviews [21, 40, 53]*.* The aim was to elicit detailed narratives of experiences and practices in the interviews by using short, open prompts and questions, and by following the thread of the interview-participant with explorative, probing questions and a minimum of interruptions. BR also tried consciously to practice active listening skills during the interviews by staying engaged and attentive throughout the whole interview [21, 40].

**Fig. 1:**
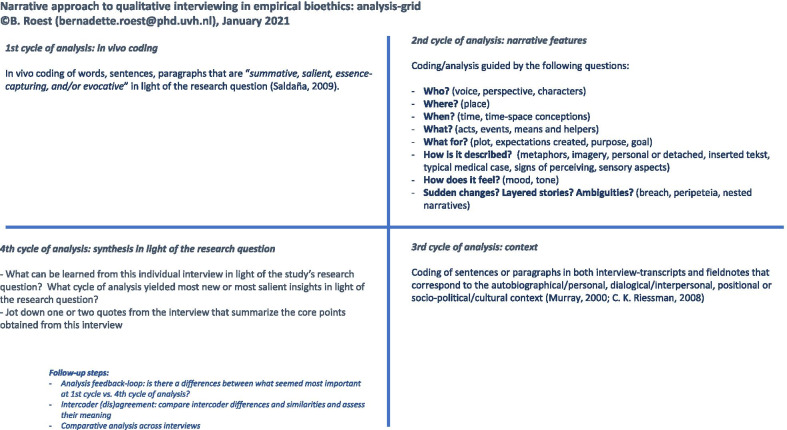
Analysis-grid

In the analysis of the interview data, BR aimed to integrate a thematic lens while paying attention to narrative features and contextual levels. The goal was to enrich the understanding of *what* is said in qualitative interviews, *how*, and in *what context*. For these reasons, BR developed a narrative approach to analyze the interview-data in different cycles and through multiple lenses as a means of avoiding some of the aforementioned pitfalls of qualitative analysis [1, 54, 55]. This approach was partly inspired by the work of Murray and Sools on narrative research [36] and of Charon et al. [14, 38] on close-reading of texts, but was adjusted to the specific research aim and setting. A grid was developed that summarizes the different cycles/lenses and that could be used as a supportive tool during analysis. See Fig. 1 for this analysis-grid.

Prior to the in-depth analysis of the interviews, all transcripts were read and re-read several times by BR and the other members of the interdisciplinary research team in order to get acquainted with the data. Preliminary thoughts, impressions and questions were jotted down. Following that, an in-depth analysis of the interviews was carried out following the different cycles presented in the grid, whereby all interviews we analyzed by BR and a subset by MM and CL.

In this stage of the research process, only one interview-encounter (including both the verbatim transcript and corresponding fieldnotes) was taken as the unit of analysis at a time. A comparative analysis of similarities and differences *between* the interviews was carried out at a later phase, a point we will address later. The first cycle of analysis consisted of an inductive process of in vivo coding of sentences and paragraphs that were seen as “summative, salient, essence-capturing, and/or evocative” in light of the research questions [55, p. 3]. If patterns of codes seem to emerge, they were organized into themes, thus resembling thematic narrative analysis [21].

The second cycle of analysis consisted of identifying and coding narrative features as described in the previous section of this article. While a multitude of narrative features could be discerned and analyzed in a text, for the sake of feasibility BR reduced her scope to the questions of ‘who, where, when, what, what for, how is it described (including metaphor), how does it feel?’ Building on insights gained from the methods of close-reading and -listening as taught in Narrative Medicine, BR also recorded unexpected twists and layered/nested stories.

The third cycle of analysis consisted of identifying and coding words and passages that referred to different levels of narrative construction as described by Murray and Riessman: autobiographical/personal, dialogical/interpersonal, positional and socio-political/cultural context [20, 21]. In this stage of analysis, attention was also paid to co-creative moments in the interviews, evidence of differences in social position between the researcher and interviewee, and possible meta-narratives or culturally shared stories present in the content (transcript) or the performance of the interview (fieldnotes).

**Fig. 2:**
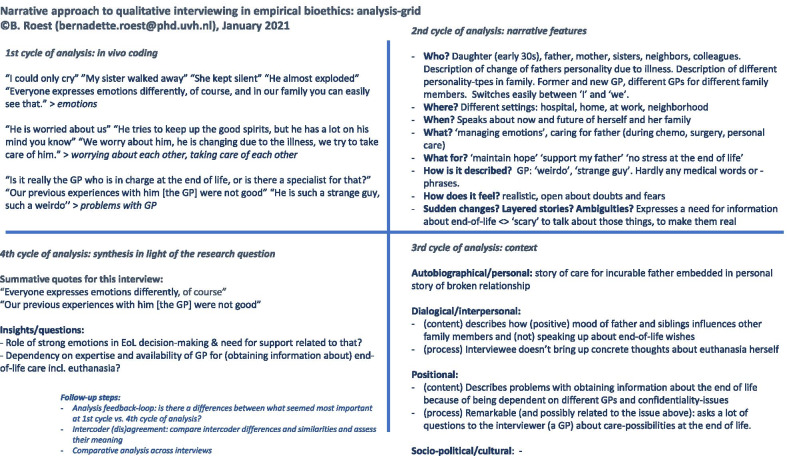
Illustration of the four cycles of analysis and the use of the analysis-grid

The fourth cycle of analysis consisted of synthesizing the results of the previous steps of analysis in light of the research questions. In addition, one or two quotes per interview were recorded that seemed to summarize the core points of that specific interview. Interestingly, two different types of quotations were often sufficient to summarize the interview: one reflecting the interviewee’s ideas with regard to the research question, and one reflecting what seemed to be off-topic with regard to the research question but nevertheless was considered very important for the interviewee. See Fig. 2 for an illustration of these four cycles of analysis and the use of the analysis-grid in this process.

After completing these four cycles of analysis for each of the interviews, the next step was a comparison and discussion of differences and similarities in the analyses of individual interviews between BR and the other researchers. Once this step was completed, BR performed a comparative analysis, first among groups of participants (i.e. the interviews of patients) and then across all the participants. This was done in order to determine whether specific themes, narrative features (such as metaphors) or contextual elements emerged among groups of participants that could shed new light on the research question. See Table 1 for an illustration of this process.

**Table 1:** Examples of close-reading the ‘Who?’ combined with cross-case analysis

*Example 1: multiple GPs instead of one* Close-reading of interviews guided by the Who?-question showed that in many cases, there was not just one GP, but many GPs involved. While the incurably ill patients ‘traveled through time and space’ towards their death, GPs retired, got sick, and went on holiday. Sometimes the patient and partner where connected to different GP offices or they were living in an area with a shortage of GPs
*Reflection* A long-lasting relationship with the GP, who knows both the patient and his/her family, is one of the cornerstones of both the practice of euthanasia and home-based palliative care in the Netherlands. The above mentioned findings challenge this cornerstone and the ideal of ‘the’ GP, although an examination of contrary-cases, scrutinizing sources of selection-bias and further (quantitative) research is warranted. The following ethical question to answer is whether this ‘multitude’ of GPs is actually a problem for ‘good’ euthanasia decision-making or not (and according to whom)
*Example 2: a change of perspective* An even closer-reading and comparative analysis of patients’ interviews revealed a remarkable change in perspective in many patients’ stories: although they spoke at length in the first person perspective (‘I want/go’ etc.), many of them changed to the second person perspective (‘you…’) when approaching the subject of the last days of their lives and the possibly hardships it may include. The same phenomenon was observed in interviews conducted as part of another empirical study about advance care planning [56]
*Reflection* To (be able to) talk openly about end-of-life preferences is an important theme in both the euthanasia- and palliative care movements in the Netherlands. The above mentioned results pose the question whether patients are capable and/or willing to talk in detail about the end of life, especially in a first person perspective. Ethically and practically, we may ask ourselves if and how we should push for open conversations about the end of life and whether another type of ACP-conversation (e.g. guided by fictional examples) may be more accessible for patients

The results of this comparative analysis were again discussed in the research team (and involved posing the question: could it have been otherwise?) and different cycles of member checks were performed (see section below). In addition, specific insights from Narrative Medicine guided the analysis and interpretation, namely (1) tolerating uncertainty and ambiguity, (2) narrative and cultural humility, and (3) focusing not only on problematic elements but also on possible strengths and sources of resilience in the stories of participants and practices being studied [13, 57, 58].

### Emerging critical insights emerging from a narrative approach

While conducting the project as described above, additional insights emerged about the possibilities and challenges of using narrative approaches in qualitative research in bioethics. In this last part of the paper, we list these insights and illustrate them with concrete examples.

#### Insight 1

The added value of training researchers through Narrative Medicine: experiencing (inter)subjectivity and practicing close-listening, close-reading and reflexivity.

While courses on social science methodology can help those without a social science background get acquainted with the application of *methods* in qualitative research, we discovered that training in Narrative Medicine (NM) teaches qualitative researchers *skills* and *attitudes* that are better aligned with an interpretive stance. In NM training, participants practice close-reading literature and artworks as well as close-listening to others’ accounts of their lived experiences [13, 14]. As BR experienced while taking NM classes, and MM observed while teaching them, immersion in Narrative Medicine activities provide opportunities to reflect on the ways in which one’s own dynamic and evolving values and personality impact all interpersonal encounters. In addition, NM activities help participants attune to details in in a patient’s story and encourage participants to practice ‘listening for the sake of listening’ rather than listening with a problem-solving or therapeutic mindset.

While the (inter)subjectivity of reading, listening and interpreting—a core tenet of the interpretive paradigm and of qualitative research—is often *explained as a concept* in methodology courses, we noticed that NM help participants e*xperience it as a practice*. For empirical bioethics researchers immersed in positivist traditions, this training can be a means of becoming sensitized to what the interpretivist traditions entail.^10^ In addition, the repeated close-reading of literature and patients’ stories in NM activities, including an analysis of storyline elements (such as voice, place, time, mood and metaphor), also constitute valuable practice for researchers interested in exploring these elements in interview data.

This awareness of (inter)subjectivity helped BR better integrate concrete reflexive elements into her project [59, 60]. For example, she discussed preliminary judgements and starting positions with the research team at the start of the research, kept a field journal throughout the whole research process (with both methodological and personal-reflective notes) and frequently debriefed with team-members and with an independent consultant (in this case, a humanistic pastoral counsellor) as a form of “third party” debriefing [43].

#### Insight 2

Sampling and conducting interviews: everyone speaks (and listens) from somewhere.

Ideas about narrative construction and the interactional context of storytelling influenced reflections in the research project about sampling and interviewing strategies. BR aimed for a heterogeneous sample of patients, relatives, GPs and other primary care professionals and chose purposive and snowball sampling techniques to recruit participants via a variety of networks (patients and relatives via both GPs and patients’ organizations, GPs and other care-professionals via both academic and professional networks), which are common practices in qualitative research.

One cannot prevent some degree of selection bias towards participants who have a special interest in the topic; this is especially true in regards to a contentious issue like euthanasia. However, the narrative approach made BR aware that more subtle forms of selection bias may be present as well; an interview in itself presupposes a certain degree of cognitive and verbal capacities on the part of the interviewee. This method of data collection can thus exclude those who are less capable of speaking in coherent stories or who may not be able or willing to read a study’s often lengthy information letter. See Table 2 for a further illustration.

**Table 2:** The cognitive task of (clinical, research) interviews

*Situation* GPs were asked to include bereaved relatives of patients with cancer who had received or who had seriously considered euthanasia. One of the GPs explicitly wrote about one of the participants that ‘she is able to verbalize well’
*Reflection* As a researcher conducting an interview-study, one may be very happy with participants who are ‘good storytellers’ as the interviews will probably yield rich data. However, some scholars in qualitative research have suggested first reflecting on the question of “what kind of socio-cognitive task” we ask of interviewees and how socio-economic status may influence that task [61]. Asking this question allowed us to reflect on whether or not a certain subset of potential participants (namely, those who could not ‘verbalize well’) or certain kinds of stories (namely, more fragmented or less coherent stories) would possibly be excluded by choosing an interview-format. This is all the more of great importance as similar questions could be asked about euthanasia decision-making itself: this process also depends to a large extent on complex ‘socio-cognitive tasks’ (i.e. deliberation with physician and family and the explication of desires, wishes and fears). Thinking about this question stimulated conversations about how we could better accommodate those who do not ‘verbalize well’ in the future, both in the practice of and inquiry into end-of-life decision-making

In addition, the narrative approach sensitized us to the role the researcher’s personality and positionality played in the interview process. While actively avoiding medical language and utterances during the interviews, BR nevertheless noticed that some interviewees frequently utilized ‘medical language’. This could have been partially due their awareness of BR’s position as both a researcher and MD. With other interviewees, BR noticed another effect of her dual role as researcher and medical professional. *Because* of her background as a MD, some participants—especially other GPs—seemed to be more willing to share personal details, which could be interpreted as a form of ‘confidence bonus’ [10]. Seen from an interpretivist paradigm, these issues are not problems that could or should be avoided. Rather, they constitute important methodological and analytical facets of the study that should be explicitly considered when interpreting the data.

#### Insight 3

Validity and research-ethics: navigating amongst interpretivist traditions.

Assessing the validity of a qualitative research project is again an undertaking that highly depends on one’s epistemological stance. While generalizabilityand internal and external validation are criteria corresponding to a positivist paradigm, narrative researchers use norms such as trustworthiness, relevance, pragmatic use and reflexivity when assessing a study [21, 40, 62–64]. Loh [62] offers a list of techniques for establishing trustworthiness; these include member checks, prolonged engagement, triangulation (of methods, sources and investigators), searching for contrasting cases, peer debriefing and thick description of research-results.

These strategies and techniques may be relatively straightforward in theory, but we want to highlight the fact that they can be difficult to apply in practice. Such techniques require a delicate dance back and forth between the interpretive and positivist paradigms, and often result in compromises when addressing methodological, research-ethical and practical issues. We encountered this tension when trying to address the ‘validation’ of our analysis grid and when we tried to conduct member-checks.^11^

We tested the grid as a tool in our narrative approach for qualitative interviewing by using it with different researchers. During this process we wondered whether it could be considered a ‘good’ tool and if it would ‘produce’ similar interpretations (cf. a positivist stance) or richer and deeper understanding (cf. an interpretive stance) amongst the various researchers. During this process, we discovered the interpretive value of both intercoder-agreement and -disagreement. We also found that discussing the tone or mood of an interview with other researchers enriched the process of analysis. See Table 3 for an illustration of this point.

**Table 3:** The interpretive value of intercoder disagreement and the ‘emotional elephant in the room’

*Situation* BR and CL both analyzed a transcript of a bereaved relative whose incurably ill parent had received euthanasia at home just four days after the patient had been discharged from the hospital because clinical specialists had concluded that there were no life-prolonging treatments left. The relative’s story spoke of a rapid physical decline in combination with a long-lasting wish for euthanasia, and agreement about that wish amongst the patient and family. While comparing our analyses, we noticed that there was agreement to a large extent in cycle 1 (in vivo codes), cycle 3 (context) and most items in cycle 2 (narrative features), although BR had jotted down more details. However, intercoder disagreement existed over the question ‘How does it feel?’ (mood, tone). Here are the answers to this question that CL and BR jotted down:
BR: Tone of disbelief. Perplexity. Laughs often but seems discordant with content of interview (comments in fieldnotes written after the interview: heaviness of everything that happened stood out. Element of surprise despite knowing what to expect)
CL: As if it all has been arranged very well, pragmatic stance of those involved, matter-of-fact approach
*Reflection* Together we examined possible explanations for the differences in perception. First, we realized that emotions easily get ‘lost in transcription’. While the interviewer (BR) sensed a perplexity and emotional gravity of the story during the interview, this couldn’t be read by CL from the transcript (despite the mentioning of non-verbal utterances). Second, we hypothesized that both interpretations may be valid, reflecting the idea of ‘tolerating uncertainty and ambiguity’. Maybe the experience was indeed ambiguous, both well-arranged given the circumstances and emotionally taxing, being remembered with both pragmatism and perplexity. Third, the different interpretations compelled both researchers to scrutinize their own perceptions: was BR (as a MD) perplexed because of this story about a ‘speedy-euthanasia’? Did CL (not being an MD) miss the impact of this speediness because he did not identify with the physician who performed euthanasia, and was more focused on the relief expressed by the interviewee? It may be counterintuitive in science—especially from a positivist perspective with its push towards objectivity—to pose the question ‘How does this feel?’, but the question yielded valuable insights about the role of emotions, rational thinking and the person of the researcher/care-professional in conversations about euthanasia

Member-check was another point of complexity. As described above, it could be seen as a *methodological* requirement given the co-constructed nature of interview-narratives. It could also be seen as an *research-ethical* requirement following the idea of the ethics of interpretation and representation—research-ethical requirements specific to narrative research apart from informed consent and confidentiality [52, 65]. That being said, details about how exactly to perform member checks—when, what and with whom—are matters of dispute, with opinions varying from merely returning the interview-transcript to participants to research-participants becoming co-analysists or co-interpretivists in the project [52, 66]

BR encountered various problems related to the process of member-checks; research-participants were not always willing or able (due to cognitive- or emotional reasons) to engage with returned research-results. Some were in a different phase of life (or even deceased) at the moment narrative summaries of the interviews were shared. Other scholars have experienced similar problems with member-checks and some have also noticed how participants may be agreeing with their own interview-narrative, but not with the interpretations stemming from a cross-case analysis [21, pp. 196–199, 67].

In her research-project, BR tried to balance the ethical requirements of careful representation and not harming participants with the methodological requirement of conducting member checks, while committing to the idea that co-construction also means that the researcher may see things that participants haven’t seen themselves or may not agree with. For example, participating patients and relatives were asked if they wanted to receive and comment on a narrative summary of their interview, but not all participants were interested in this offer. In order to ensure that patients’ and relatives’ perspectives were still being accurately represented in the data, BR also asked representatives of patient-federations to provide feedback on the interview-data as summarized in draft articles.

On a final note, the trustworthiness and relevance of this project will ultimately be determined by the broader professional and scholarly community as “validity rests on a consensus within a community of speakers” [63, p. 474]. This implies that a researcher be able to address and ‘speak the language’ of these various communities.

#### Insight 4

From the empirical to the normative: the moral work of choosing methodologies

The final step in an empirical bioethics project typically entails linking the empirical results to normative conclusions. In the case of BRs research project, the goal was to critically explore and ultimately inform the ethical evaluation of euthanasia decision-making in general practice. Following Walker and other ethics scholars who have written on narrative approaches, we take the stance that narratives—or any empirical data—cannot ‘speak for themselves’ as leading to normative conclusions. In other words, coherent and authentic narratives cannot serve as a substitute for moral justification [33, 47, 68]. Walker [33, p.13] argues for a “fully normative reflection” which could be assessed with criteria of good philosophical argumentation [10, 69].

While BR is still in the process of evaluating the empirical results at the time of writing this article, one issue that comes to the fore is the need to rethink the question of ‘plot’: what is the underlying purpose or goal of euthanasia, and according to whom? While GPs may frame it as a last resort in case of untreatable symptoms when death is imminent, patients and relatives may speak of it as a possibility to avoid the dying process and accompanying physical deterioration and dependency, or merely as ‘one more way of dying’. In addition, a detailed look at the who, where and when in the stories about both euthanasia and palliative care in the home-care setting raises questions about access to and availability of professional care in theory versus practice. Furthermore, a close-reading of metaphors helps one reflect upon different perceptions of care-receivers versus -providers in regards to equality, dependency and the role of emotions in end-of-life decision-making. These issues will be discussed in an upcoming publication.

In this article, our main goal was to draw attention to the ‘normative work of choosing methodologies’. Like Mertz et al., we contend that it is an important *ethical* task to scrutinize the methodologies we use, because “poor methodology in EE may give rise to misleading ethical analyses, evaluations or recommendations, not only depriving the study of scientific and social value, but also risking ethical misjudgment” [10]. Or to use a medical analogy: if one only uses an ophthalmoscope (i.e. an instrument to look into the eyes), one will never hear the heartbeat.

Finally, our thorough consideration of methodology has also lead us to reflect critically about the purported aims and ambitions of empirical bioethics. With this article and corresponding methodological approach, we want to stress the importance of what we call the ‘diagnostic task’ of empirical bioethics. In other words, we actively tried to conduct an open-ended examination of moral dimensions that were previously not in the scope of ethicist or that may be framed or perceived differently in practice [70]. In our view, this also includes considering issues such as epistemic (in)justice in medicine and biomedical or bioethical research [25, 71, 72] and further theorizing about the subject of listening and how that relates to bioethics and broader, to democratic practices [73]. We intend to explore these issues in future publications as well.

## Conclusions

In this article, we have discussed the challenges one may face when conducting qualitative interviews for bioethics research as well as the tensions related to positivist and interpretivist traditions in science. We have shown how insights from narrative research and Narrative Medicine could enrich qualitative interviewing in medicine and bioethics by deepening our understanding of the co-construction and situatedness of stories (whether in the clinic or in research-encounters) and the importance of (inter)subjectivity and positionality.

We do not consider these insights and corresponding approach to be a substitute for positivist approaches in bioethics and medicine, rather as a necessary complement. We also realize that “fancy epistemological footwork” may be required when one tries to combine different traditions and corresponding methods in one’s research project or department [51, p. 706]. In addition, narrative approaches to conducting and analyzing qualitative interviews may be time-consuming and labor-intensive. However, we think that some topics in bioethics require ‘slow-reading’ and profound reflection in order to grasp the nuances and particularities of the topic, and to formulate ethical guidance that is in-tune with the utterances of those who will effected by such guidance.

Furthermore, we realize that, “any methodological standpoint is, by definition, partial, incomplete and historically contingent” [51, p. 706], and therefore hope to engage in further conversations on the issues foregrounded in this article. We are currently trying to further develop narrative approaches in the field of empirical bioethics, and are exploring the possibility of integrating ethnographic approaches^12^ with insights from cultural/socio-narratology and narrative ethics. [76–80]

Last, we shared some of our ongoing process of considering and scrutinizing meta-ethical and methodological positions, which we see not just as a *practical* but as an *ethical* task that influences the course and outcome of one’s empirical bioethics project. We think that bioethics could benefit more from approaches that foster close-listening and close-reading of people and practices in healthcare, thereby enabling more ‘diagnostic thinking’, an attitude we consider to be a prerequisite for formulating ethical guidance. Whether our narrative approach will be useful to others in the field, is a question that will have to be answered in the future. In the meantime, our work can already serve as a valuable contribution to ongoing discussions about the place of interpretivist traditions in empirical bioethics that entail a different view on knowledge-generation and subsequently on quality criteria for validation, reporting, research-ethical evaluation and training for researchers.

## Data Availability

Not applicable.

## Abbreviation

GP: General practitioner

## Footnotes

1 We use ‘narrative approaches’ as an umbrella term for the work done by a broad group of scholars in diverse academic disciplines and professions (sociology, anthropology, literature studies, ethics, healthcare, social work, psychotherapy and more) who all focus on storied forms of knowledge marked by (inter)subjectivity, contingency and temporality. Nevertheless, a myriad of research methods and theoretical assumptions can be found among these different scholars. See Spector–Mersel [1] for an interesting discussion of this diversity and commonalities among narrative researchers and the historical background of the ‘narrative turn’. See Squire [2], Greenhalgh [3] and Nelson [4] as well for background information about narrative approaches in the social sciences, healthcare and bioethics respectively.

2 We define ‘empirical bioethics’ as the broad interdisciplinary academic field (involving health professionals, ethicists, social scientists and scholars with other backgrounds) dedicated to addressing normative questions related to healthcare practices through empirical inquiry and theoretical reflection.

3 Narrative Medicine is the phrase used by Charon and colleagues for their clinical-educational literature and medicine program that has taken shape at Columbia University NYC since the start of this millennium [13, 14]. See the article of Charon et al. [14] for a summary of their pedagogy of “teaching attention, representation and affiliation” through the study of literature and artworks (close-reading) and creative writing, and its theoretical underpinnings. This article also includes a “Reading guide for reflective practice” that illustrates how one could examine a text following concepts such as ‘form’, ‘voice’ and ‘mood’. See Jones [15] and Crawford et al. [16] for comprehensive background information about the role of narrative and literature in the medical humanities in the past three decades.

4 Interpretivism is used as an umbrella term for epistemologies marked by a view on reality as “multifaceted (..), exchanging the positivist desire to create a ‘true’, neutral and unbiased picture of the ‘actual’ reality for an emphasis on the subjective component inherent in the study of social reality” [1, p. 209]. For the sake of clarity, we have somewhat simplified the question of epistemology by using two poles of positivism and interpretivism. However, different types of interpretivist traditions can be discerned (see Spector–Mersel, 2010 [1]) and other epistemologies such as critical theory or pragmatism could also inform qualitative and narrative research.

5 Experienced bioethics researchers may easily move along the spectrum of paradigms and concordantly different views on what kind of results qualitative research may engender. However, we have noticed that this is less easy for (young) healthcare professionals who have just started immersing themselves in bioethics and/or qualitative research. To provide one example, in a third-year required course on Medical Humanities at the UMC Utrecht, one of the goals is to help students become more aware of their norms and values, and to situate utterances like these in broader philosophical, social and cultural contexts. At the onset of the course, students must write a short essay answering the question “what is science” and describing what they consider the definition and purpose of scientific research to be. The overwhelming majority of essays in MM’s groups are positivistic in stance and content, for example defining science as “reliable and not subjective”.

6 Again, this is a simplification of theoretical standpoints for the sake of clarity in this article. See Squire et al. [2] for a nuanced discussion of “theoretical divisions in narrative research”, as well as Spector-Mersel [1] for a discussion about “factist” versus “discursive, constructivist or postmodern” views on narratives.

7 See Vaismoradi et al.[34] for an in-depth discussion of thematic and content analysis and Hardy et al. [35] for a comparison of content analysis and discourse analysis.

8 This article confines itself to a discussion of narrative approaches that are common in biomedical fields (including the related disciplines of health psychology and social work). We are well aware that insights from the field of narratology could further enrich our thinking about narrative approaches, see our comments in the conclusion.

9 According to many narrative scholars including Riessman, the mode of transcription is already an important part of analysis and interpretation. See McLellan et al. [50] for useful background information about and guidelines for transcription.

10 In conversations about this topic, BR and MM spoke about the ways in which NM activities helped ‘expand the frame of scientific thinking’ that BR as a positivist-minded physician/researcher initially operated from. We don’t want to imply that this effect cannot be reached by taking methodology courses or being mentored by experienced qualitative researchers. However, BR was surprised by how NM activities fostered a deeper understanding of (inter)subjectivity than the various methodology courses she had previously followed.

11 Other emerging research-ethical issues will be discussed in a separate publication.

12 A narrative approach to qualitative interviews proved to be most suited to BR’s research project, but we certainly think that the study of ‘enacted stories’ [3] could further enrich our understanding of (moral) life in healthcare. MM is currently conducting empirical research on the digital transition in pathology and radiology for the RAIDIO study (Responsible Artificial Intelligence in clinical DecisIOn-making). As part of the project, interviews and field observations will be used to develop and publically screen an ethnographic film about the use of AI in image-based medicine as a means of generating public debate and reflection on the digitalization of image-based health care [74, 75].
